# Stress and musculoskeletal pain in physiotherapists during the pandemic depend on a plethora of influencing factors

**DOI:** 10.1186/s12998-023-00526-9

**Published:** 2024-02-16

**Authors:** Josef Finsterer, Carla Alexandra Scorza, Fulvio Alexandre Scorza, Antonio-Carlos Guimaraes de Almeida

**Affiliations:** 1 Neurology Department, Neurology and Neurophysiology Center, Postfach 20, 1180 Vienna, Austria; 2 Disciplina de Neurociência, Universidade Federal de São Paulo/Escola Paulista de Medicinal (UNIFESP/EPM), São Paulo, Brazil; 3 Centro de Neurociências e Saúde da Mulher “Professor Geraldo Rodrigues de Lima”, Escola Paulista de Medicina/Universidad Federal de São Paulo (EPM/UNIFESP), São Paulo, Brazil

**Keywords:** Musculoskeletal pain, Depression, Stress, SARS-CoV-2, Pandemic

**Letter to the Editor**

The interesting article by Weiss et al. is impressive, but several points require discussion [1].

The main limitation is that only a small spectrum of factors influencing the development of psychosocial stress and musculoskeletal pain were included in the analysis. Because the range of influencing factors is much broader, the results provided may be misleading and may not reflect real world experiences.

First, the type of patients treated by the physiotherapists was not specified. Psychological distress and musculoskeletal pain can highly depend not only on the physical activity in general, but also on the type/intensity of activity required for specific patient groups during work. Treating a patient with severe stroke may be more stressful than treating a patient with tension-type headache.

Second, sleep duration and quality were not included in the analysis. Dealing with stress heavily depends on the degree of relaxation during sleep. The more relaxed a person is after sleeping, the better able they are to process everyday environmental stress.

Third, leisure-time physical activity was not included in the assessment. Depending on the type and intensity of physical activity during leisure time, musculoskeletal pain can be more or less severe. Therapists who regularly participate in endurance sports may react with different level of stress/pain than those who regularly play minigolf.

Fourth, attitudes toward the job and working conditions were not assessed. Those who are satisfied with their working conditions may react psychologically and physically differently than those who are dissatisfied with them.

Fifth, the experience of stress and musculoskeletal pain also depends heavily on the working hours and ultimately the number of overtime hours of each participant. It is quite likely that those with a higher number of working hours suffer more psychological stress and musculoskeletal pain than those with a low number of working hours.

Sixth, the results may also depend heavily on the work experience and coping mechanisms of the participants included. People with long-term work experience may cope with working conditions and circumstances during the pandemic differently than those with a short-term experience.

A limitation of the study is that no control group was examined. To answer the question of whether psychological stress and musculoskeletal pain have really increased during the pandemic, a control group matched for age, gender, type of treated patients, type and intensity of leisure time physical activity, working hours, experience, and attitude towards the job analysed before the pandemic must be compared with patient group.

## Data Availability

All data are available from the corresponding author.
